# Mechanisms of ATP Release by Inflammatory Cells

**DOI:** 10.3390/ijms19041222

**Published:** 2018-04-18

**Authors:** Michel Dosch, Joël Gerber, Fadi Jebbawi, Guido Beldi

**Affiliations:** 1 Department for Visceral Surgery and Medicine, Bern University Hospital, University of Bern, CH-3010 Bern, Switzerland; michel.dosch@dbmr.unibe.ch (M.D.); joel.gerber@students.unibe.ch (J.G.); fadi.febbawi@dbmr.unibe.ch (F.J.); 2 Department for BioMedical Research (DBMR), Bern University Hospital, University of Bern, CH-3008 Bern, Switzerland

**Keywords:** ATP release, extracellular nucleotides, purinergic signaling, inflammation, monocytes/macrophages, neutrophils, endothelial cells, non-specific nucleotide release, vesicular exocytosis, connexins, pannexins

## Abstract

Extracellular nucleotides (e.g., ATP, ADP, UTP, UDP) released by inflammatory cells interact with specific purinergic P2 type receptors to modulate their recruitment and activation. The focus of this review is on stimuli and mechanisms of extracellular nucleotide release and its consequences during inflammation. Necrosis leads to non-specific release of nucleotides, whereas specific release mechanisms include vesicular exocytosis and channel-mediated release via connexin or pannexin hemichannels. These release mechanisms allow stimulated inflammatory cells such as macrophages, neutrophils, and endothelial cells to fine-tune autocrine/paracrine responses during acute and chronic inflammation. Key effector functions of inflammatory cells are therefore regulated by purinergic signaling in acute and chronic diseases, making extracellular nucleotide release a promising target for the development of new therapies.

## 1. Introduction

Cellular respiration in the mitochondria converts nutrition-derived energy into adenosine 5′-triphosphate (ATP), the usable form of chemical energy in the body. Although most of the ATP (and other nucleotides) is located intracellularly, it is released into the extracellular space under specific conditions, where it is a relevant signaling molecule. The importance of ATP in inflammation has been established in the last decades [1,2]. In particular, ATP released from stressed cells functions as a “danger” and “find-me” signal for phagocytes to migrate to the damaged tissue [3,4]. In addition, ATP provides qualitative and quantitative information about pericellular injury to inflammatory cells via autocrine/paracrine purinergic signaling. Thus, nucleotides can define how and to what extent inflammatory cells should react through interaction with specific receptors [5]. Such P1 and P2 receptors are conserved germline encoded pattern recognition receptors (PRRs). In other words, during inflammation, ATP is released from inflammatory cells—and parenchymal cells—to fine-tune their activation via autocrine/paracrine signaling.

Several specific properties make extracellular purines and pyrimidines efficient damage signaling molecules. First, because of their rapid release via vesicular exocytosis or pores upon cell activation. Second, because ATP is water-soluble and freely diffusible in the extracellular environment due to negatively charged phosphate residues. In addition, third, a high concentration gradient between extracellular and intracellular compartments (micromolar compared to millimolar range, respectively) creates a strong signal to noise ratio with very low background noise [6].

The focus of this review is on the mechanisms of ATP release from inflammatory cells. Key publications cited in this work are summarized in Table 1. Given the importance of purinergic signaling in modulating the inflammatory response, it is crucial to understand the way ATP is released into the extracellular space. Such understanding may be clinically relevant since blocking ATP release specifically may modulate the whole purinergic signaling cascade in an upstream manner and thereby offers new avenues for drug therapies [7].

## 2. Non-Specific ATP Release from Necrotic Cells

Upon inflammation, cell membrane disruption leads to non-specific release of large amounts of nucleotides due to the high intracellular nucleotide content compared to the extracellular space [4,8]. ATP released from necrotic parenchymal cells in damaged tissue is then recognized by inflammatory cells. As an example, sepsis is associated with organ damage remote from primary infection site because of a systemic inflammatory reaction syndrome caused by infection [9]. Specific and non-specific release of large amounts of ATP are observed during sepsis [10], which then fine-tune the activation of inflammatory cells in an autocrine/paracrine manner. Enzymatic degradation of systemic ATP by ectonucleotidases during sepsis has been shown to improve bacterial clearance and prolong survival [11,12]. Application of apyrase, an ecto-ATPase, prevents the accumulation of IL1 β and other inflammasome-dependent cytokines (TNF α, IL10) in caecal ligation puncture (CLP), a peritoneal sepsis mouse model [12]. ATP hydrolysis by apyrase additionally promotes polymorphonuclear neutrophils (PMNs) chemotaxis, leading to improved host defenses [11]. 

Furthermore, extracellular ATP serves as a “find-me” signal to attract phagocytes, that mediate the clearance of necrotic bodies [13]. The P2Y2 receptor senses ATP but also UTP released from necrotic cells and seems to be relevant for necrosis detection by neutrophils and macrophages [5,13]. 

Taken together, ATP release by necrotic cells generates a pro-inflammatory microenvironment via pro-inflammatory cytokines secretion and recruitment of neutrophils to the site of necrosis [4,8,14].

## 3. Active ATP Release via Vesicular Exocytosis

**Figure 1:**
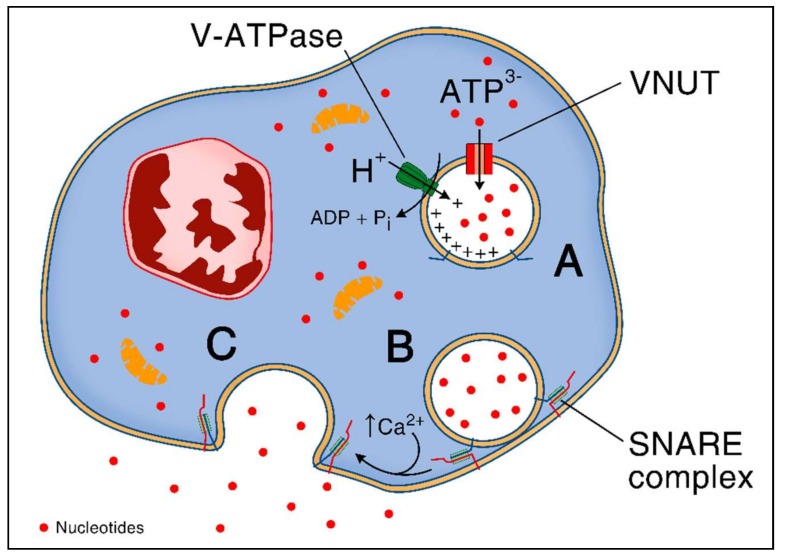
ATP release through vesicular exocytosis. (**A**) Active transport of ATP inside the vesicles through the vesicular nucleotide transporter (VNUT) using V-ATPase generated proton gradient (positive inside the vesicle) as a driving force; (**B**) SNARE zippering occurring spontaneously; (**C**) Increased intracellular calcium concentration leads to SNAREs mediated membrane fusion and release of ATP into the extracellular space.

Specialized cytosolic granules store and then rapidly release ATP via fusion with the plasma membrane [15,16]. Storage and exocytosis of ATP depends on the vesicular nucleotide transporter (VNUT) which is also responsible for subsequent exocytosis of divalent cations [17]. This protein is a secondary active transporter, ensuring the accumulation of cytosolic ATP inside the secretory vesicles in a chloride-dependent manner. It uses an electrochemical proton gradient (positive inside the granules) generated by V-ATPase as a driving force [17,18]. VNUT protein is encoded by SCL (solute carrier) *17A9* gene, which is a member of the SCL17 family of ion transporters that are widely expressed among vertebrates and non-vertebrates [17,18]. After transfer of ATP into secretory granules, the mechanism of vesicular exocytosis has been shown to depend on intracellular Ca^2+^ levels and SNARE (soluble N-ethylmaleimide-sensitive-factor attachment receptor) family of proteins [19]. SNARE proteins mediate membrane fusion via the interaction of *v*- and *t*-SNARE subtypes that zipper up and form the *trans*-SNARE complex, an α-helical bundle, that pulls the two membranes tightly together as shown in macrophages [20]. The mechanism of ATP release through vesicular exocytosis is summarized in Figure 1.

### 3.1. Vesicular ATP Release in Response to Inflammation Mediates Chronic Inflammatory Pain

Perception of pain can be a consequence of inflammation and is directly modulated by extracellular ATP [38,39,40]. In particular, ATP is released during tissue damage and activates indirectly nociceptors on neurons, which are expressing P2 receptors [41]. Blocking of vesicular exocytosis-mediated ATP release from neurons, microglia and immune cells using clodronate, a bisphosphonate, reduces chronic inflammatory pain in mice [21]. Downstream, ATP-mediated nociception occurs via the activation of P2X7 on microglial cells leading to production and release of inflammatory cytokines [42]. These results are supported by the clinical observation that bisphosphonates reduce pain when they are used to treat bone metastases from breast or prostate cancers [43].

### 3.2. Vesicular Exocytosis-Mediated ATP Release in Response to Infection

Pathogen associated molecular patterns (PAMPs) released during infection induce active ATP release from inflammatory cells. In response to unspecific (ionomycin) or Toll-like receptors 4 (lipopolysaccharides, LPS) and 2 (Pam3CSK4) stimuli, vesicular exocytosis-mediated ATP release was observed for microglia, bone marrow and peritoneal macrophages, and THP-1 monocytes [19,35,37]. Only blockade of vesicular exocytosis by N-ethylmaleimide (NEM), but not hemichannel blockers (carbenoxolone, probenecid or flufenamic acid) inhibited LPS-induced ATP release by RAW264.7 macrophages [35]. However, other studies observed nucleotide release via connexin-43 and pannexin-1 under similar conditions [22,27]. These discrepancies might underline parallel contribution of both vesicular exocytosis and hemichannels in LPS-induced ATP release by monocytes/macrophages. Differences might further depend on particular experimental settings, including cell types tested and kinetics of ATP measurements. Once in the extracellular space, ATP released through vesicular exocytosis regulates THP-1 monocytes activation in an autocrine manner via P2Y11 activation [37]. In addition to LPS, viruses and their products can also mediate vesicular ATP release. In particular, vesicular stomatitis virus (VSV) infected macrophages release ATP via both vesicular exocytosis and hemichannel pannexin-1 [25].

Vesicular release of ATP, which is Ca^2+^-dependent, can further induce changes in cytosolic calcium concentration by the activation of P2Y receptors in an autocrine manner [44]. Indeed, treatment of monocytes using NEM or P2Y receptors antagonists blocks intracellular Ca^2+^ increase following lysosomes disruption, which play a critical role for monocytes functions, including responses to LPS, for chemotaxis, phagocytosis, and cytokines production [45,46,47,48,49].

### 3.3. Vesicular Exocytosis Mediated ATP Release in Response to Hypoxia

It is accepted that hypoxia leads to inflammation by inducing production of inflammatory cytokines through activation of hypoxia-inducible factors (HIF) [50]. HIFs amplify the NFκB pathway, among others by increased expression and signaling of Toll-like receptors (TLRs) [51]. Alternatively, hypoxia induces inflammation through ATP release [52,53,54]. Indeed, hypoxia induces ATP release from endothelial cells by vesicular exocytosis, a mechanism dependent on phosphoinositide 3-kinases (PI3K) and Rho-associated protein kinase (ROCK) [28]. Released ATP induces venous dilation via increase in prostaglandins and nitric oxide (NO). In this study, autocrine purinergic signaling decreased in endothelial cells upon hypoxia limiting P2 receptor-mediated [Ca^2+^]_i_ increase and self-regenerating ATP release via vesicular exocytosis in a negative feedback loop [28].

## 4. Active ATP Release via Pore-Forming Channels

### 4.1. Connexins and Pannexins—Structural and Functional Differences

In addition to vesicular exocytosis, inflammatory cells release nucleotides in the extracellular space via connexin hemichannels or pannexin channels, mainly connexin-43 and pannexin-1 [5]. One critical remaining challenge in the field is to distinguish their respective contributions [55]. The main functional difference between these two types of hemichannels is that connexin proteins can form both gap junctions and hemichannels, while pannexin proteins only form (hemi)channels [56]. Even though pannexins do not share sequence homology with connexins, both proteins display N- and C-terminal domains in the cytoplasm, four membrane spanning segments and extracellular and intracellular loop domains [57,58]. Connexins and pannexins are both four-pass transmembrane proteins that assemble to form a hexameric structure called connexon and pannexon respectively. While connexins contain six *Cys* residues in their extracellular loops, pannexins contain four and a consensus sequence for glycosylation [56,57]. Connexons and pannexons mediate the release of small molecules in the extracellular space, including ATP, glutamate, prostaglandins and others typically below 1–2 kDa, as well as the influx of ions such as Na^+^ and Ca^2+^ [59,60]. Importantly, both channels were shown to be permeable to ATP in opened state and to mediate its release [61,62]. In addition to connexin-43 and connexin-37 (see below), other connexin isoforms, including connexins-26 and -36, were shown to mediate ATP release into extracellular space [63]. Epithelial cells from the colonic mucosa release ATP via connexin-26 upon *Shigella flexneri* infection, the intracellular causative agent of bacillary dysentery that elicit an intense inflammatory reaction [64]. Connexin-26 opening and subsequent ATP release are dependent on actin and phospholipase-C. This mechanism contributes to bacterial invasion and spreading. Connexin-36 mediates ATP release from depolarizing neurons [65]. Other connexin channels release ATP in other cells than inflammatory cells and in other contexts than inflammation [63].

Gating of both connexons and pannexons is regulated by changes in transmembrane voltage (Vm) [66], extracellular or intracellular calcium concentrations [67,68], mechanical strain or post-translational modifications [63]. The specific mechanisms for each channel subtype are detailed below in Section 4.2 and Section 4.7 respectively.

### 4.2. Structure and General Functions of Connexin Hemichannels

**Figure 2:**
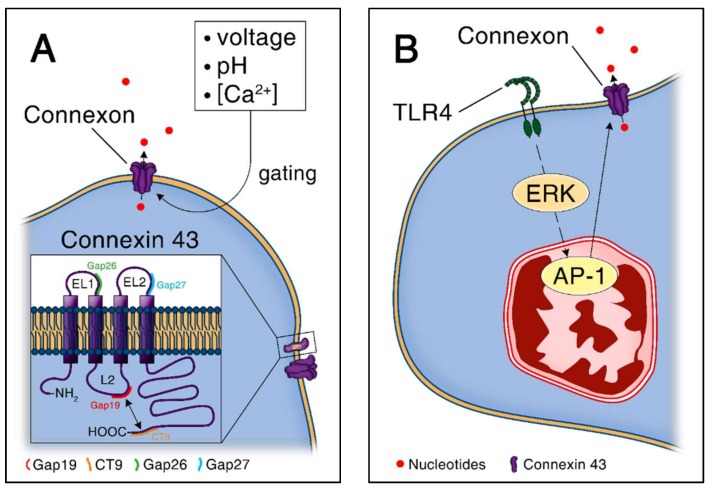
ATP release through connexin-43 hemichannels. (**A**) Connexin-43 gating and structure. Gap26 and Gap27 are connexin-43 specific blockers that target extracellular loops, whereas Gap19 and CT9 target intracellular loops. Loop-tail interactions are represented by a double arrow; (**B**) Connexin-43 expression is induced in response to Toll-like receptor 4 agonist (LPS) and is dependent on ERK/AP-1 signaling in macrophages.

Connexin proteins contribute to various cellular and physiological functions by forming gap junctions or unopposed hemichannels, which allow intercellular communication. For convenience, connexins are classified according to the molecular weight of their subunits. To date, 21 connexin isoforms are identified in the human and 20 in the mouse genome, among which connexin-43 is the most widely expressed [69,70]. Under homeostatic conditions, hemichannels are more likely to be in closed rather than in an open state to avoid the loss of vital ionic, energetic, and metabolic gradients [71,72]. However, connexin hemichannels are opened by electrical, chemical and mechanical stimuli as shown in Figure 2A [72]. The regulation of the opened/closed state of connexin-43 was shown to be dependent on the interaction between its cytoplasmic loop and cytoplasmic tail [72,73]. In particular, interactions between the last nine amino acids at the end of the cytoplasmic tail of the connexin-43 protein and the L2 domain, located on the cytoplasmic loop, determine opening of the hemichannel [73]. This mechanism is further dependent on intracellular calcium concentrations and positive cell membrane voltage changes [74]. In addition, connexin-43 phosphorylation at multiple serine residues induces conformational changes that determine hemichannel oligomerization and subsequently the formation of hemichannels or gap junctions [75,76,77]. Trafficking to or within the plasma membrane, hemichannel opening and ultimately connexin degradation are also related to phosphorylation events [75,76,77]. Reactive oxygen species, typically released in large amounts during inflammation, modulate connexin hemichannels via oxidation of several potentially oxidizable amino acids and nitric oxide-mediated *S*-nitrosylation of cysteine residues [78]. Especially, redox-mediated regulation of connexin proteins can lead to connexin-43 hemichannels opening [79]. There are specific inhibitors targeting the extra- and intra-cellular mechanisms. These include mimetic peptides derived from highly conserved regions, including Gap26 and Gap27 that target the first and second extracellular loop of connexin-43, respectively [72]. Gap19 is a nonapeptide that targets the L2 domain with the advantage to block specifically connexin-43 hemichannels and not gap junctions [80].

### 4.3. Connexin-Mediated ATP Release in Response to Pathogen Associated Molecular Patterns

In monocytes and macrophages, TLRs activation is associated with connexin-43 expression. LPS from *Escherichia coli*, a TLR4 agonist, and Pam3CSK4, a TLR2 agonist, lead to connexin-43 expression in an ERK/AP-1 dependent manner in a macrophages cell line (RAW 264.7), as presented in Figure 2B [27]. This results in the release of UDP and ATP from macrophages that activate purinergic receptors such as P2Y6 by UDP and P2X7 by ATP [27,34]. In an autocrine loop, UDP induces MCP-1 release from macrophages via P2Y6 activation, a mechanism that seems to protect from *Escherichia coli* bacteremia [27]. In parallel, ATP activates P2X7 and increases inflammatory cytokine levels and bacterial load [34]. Systemic pharmacological blockade of connexin-43 by Gap 27 led to increased levels of inflammatory cytokines IL6 and IL10 in the peritoneal lavage after caecal ligation and puncture [34]. However, outcome of peritoneal sepsis upon connexin-43 blockade was not assessed in this study.

PMNs play a critical role in the acute phase of the inflammatory reaction to PAMPs beside monocytes/macrophages. PMNs have been shown to release ATP via connexin-43 upon activation with *N*-formyl Met-Leu-Phe (fMLP), a potent PMNs chemotactic factor, and LPS [24,81]. This was tested by treating PMNs using Gap19 and in PMNs partly or totally deleted for connexin-43. Mechanistically, the ATP released through connexin-43 by PMNs in response to LPS stimulation activates P2X1 receptors leading to intracellular calcium increase and eventually to myosin light chain activation [24]. This mechanism serves as a “stop signal” for neutrophils in order to halt their migration at the infectious foci [24].

Endothelial cells are another important cell type in the regulation of inflammation to PAMPs via connexin-43-mediated ATP release. Indeed, ATP release through connexin-43 hemichannels occurs from endothelial cells in response to TLR2 agonists, more precisely peptidoglycans derived from *Staphylococcus epidermidis*, a process that further initiate an inflammatory reaction [82]. In addition, human microvascular endothelial cells (HMVEC) were shown to release ATP through connexin-43 hemichannels in response to foreign material [33]. The implantation of foreign material is a potential trigger of sterile inflammation, a process called foreign body reaction, and purinergic signaling contributes to the early inflammatory events of this reaction [83].

### 4.4. Connexin-Mediated ATP Release in Atherosclerosis

Purinergic signaling plays a critical role in the pathophysiology of atherosclerosis [84,85]. In this context, inflammation-induced adhesion of monocytes to the endothelium is a crucial event that is modulated by connexin-43-mediated ATP release from monocytes [86]. Released ATP is rapidly converted to adenosine (ADO) by ecto-ATPases, which decreases the adhesion of circulating monocytes to endothelium, a process that prevents the formation of atherosclerosis [87]. In an older study, a macrophage cell line (H36.12j cells) transfected with connexin-37, another connexin isoform, released higher levels of ATP [88]. These results are of interest in atherosclerosis pathogenesis, since deletion of connexin-37 gene in apolipoprotein E-deficient mice (Apoe^−/−^, a mouse model of atherosclerosis) leads to increased aortic lesions compared to conventional Apoe^−/−^ mice [88]. Investigations using purinergic agents for the treatment of atherosclerosis are in progress with potential for new therapeutic targets [7].

### 4.5. Hypoxia Regulates Connexin-Dependent ATP Release

Connexin-43 expression and subsequent ATP release in vascular endothelial cells were shown to decrease under hypoxic conditions [89]. Specifically, hypoxia resulted in connexin-43/ser368 phosphorylation, a process that induces a conformational change in the channel associated with decreased channel permeability [77]. Conversely, during intestinal neuro-inflammation, hypoxia induces damage and oxidative stress in the gut. Connexin-43-mediated ATP release by local glial cells upon hypoxia increases damage by inducing neuro-inflammation, which kills enteric neurons and contributes to the development of motility disorders [26]. As shown above, alterations of hypoxia regulate ATP release via vesicular exocytosis and connexin hemichannels and seem to be highly context-dependent as well as insufficiently understood.

### 4.6. Regulation of Pannexin Channels

**Figure 3:**
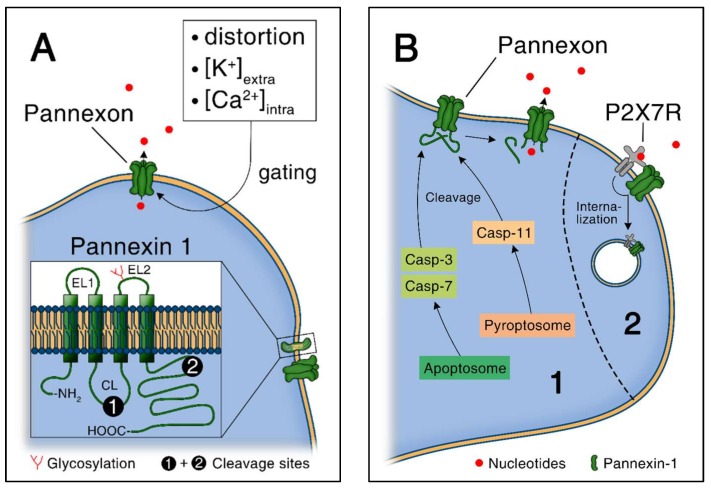
ATP release through pannexin-1 channels. (**A**) Pannexin-1 gating and structure. Caspase cleavage sites are located on the intracellular loop and the C-terminal tail; (**B**) Pannexin-1 channels activation via caspase-mediated cleavage [1]. The pore is plugged under homeostatic conditions and opens upon cleavage. Upon higher extracellular nucleotide concentration, activation of P2X7 receptor (P2X7R) leads to internalization of Pannexin-1 in terms of an autocrine negative feedback loop.

Pannexin proteins are orthologues of invertebrate innexins and consist of 3 members, including pannexin-1, -2 and -3 [57,63]. Pannexin-1 and -3 are widely expressed, whereas pannexin-2 is mainly expressed in the brain [59]. Here, the focus is on pannexin-1 due to its function in inflammation [59]. Like connexin hemichannels, pannexin channels are likely to stay closed under homeostatic conditions. Concretely, the C-terminal tails of pannexin-1 proteins maintain the channel closed, presumably by directly plugging up the pore from the intracellular side as shown in Figure 3A,B [71]. The cleavage of the C-terminal tail of pannexin-1 proteins by activated caspases, including caspase-3 and -7 during apoptosis and caspase-11 during pyroptosis and endotoxic shock, plays a critical role in pannexin-1 channel activation [31,90,91]. The cleavage of successive C-terminal tails inside the pannexon is associated with a progressive opening of pannexin-1 channel and an increase of the permeability to ions and larger molecules including nucleotides [92].

Altogether, various triggers lead to opening and activation of pannexin-1 channels, including mechanical stress [62] and increased intracellular calcium or extracellular potassium [68,93]. Ligand-gated receptors activate pannexin-1 channel for ATP release as well, including ATP-gated ionotropic P2X7 receptor [94,95,96], protease activated receptor 1 (PAR-1) receptors following thrombin stimulation [97], H1 receptors in response to histamine [98] and B2 receptors in response to bradykinin [99]. Also redox potential changes could play a role [100].

In an autocrine negative feedback loop, pannexin-1 channels can be inhibited by ATP as shown in Figure 3B [101,102]. In particular, ATP released through pannexin-1 channels interacts with P2X7 receptors and mediates the internalization of pannexin-1 channels in a concentration- and time-dependent manner in Neuro2a cells [101]. In contrast to connexin-43, phosphorylation of pannexin-1 proteins as a mean to modulate channel functions have not been studied in depth yet [103]. However, one study is showing that NO attenuates pannexin-1 channel function by phosphorylation of a serine residue through a cGMP-PKG dependent pathway in HEK-293 cells [104].

### 4.7. Pannexin-1 Mediated ATP Release from Apoptotic Cells

In contrast to necrotic cells, apoptotic cells release ATP in a controlled manner, via pannexin-1 channels [90,105]. The role of ATP and UTP as damage associated molecular patterns, “danger signals” that guide inflammatory cells to the site of injury, is a well-established concept [3,14,106]. In particular, it has been shown that ATP and UTP released during apoptosis attract monocytes to remove cellular debris [14,107]. These “find-me” nucleotides are specifically released from apoptotic cells via pannexin-1 channels after caspases-3 and -7 mediated cleavage as shown in Figure 3B [90]. ATP released from apoptotic cells is not only important for phagocyte recruitment, but also for the secretion of IL1 β by macrophages via activation of Nlrp3 inflammasome [105].

### 4.8. Functional Consequences of Pannexin-1 Mediated ATP Release during Inflammation

#### 4.8.1. Neutrophils

Proper chemotaxis of neutrophils requires an excitatory signal at the front edge and an inhibitory signal at the rear of the cell [108]. ATP released via pannexin-1 channels mediates neutrophil activation at the front edge via P2Y2 receptor autocrine stimulation [109]. In addition, pannexin-1 channels also provide inhibitory signals at the rear of the cell by releasing ATP, which is further degraded to adenosine by CD39 and CD73 [108]. Finally, adenosine signals via adenosine A2a receptors that redistribute to the rear of the neutrophil and block chemoattractant receptor signaling [30,110].

#### 4.8.2. Monocytes/Macrophages

In macrophages, synthesis and secretion of IL1 β is dependent of pannexin-1 mediated ATP release. The process requires endogenous ATP secretion via pannexin-1 channels by macrophages in response to LPS and the consequential interaction with P2X7 receptor for the assembly of oligomerized Nlrp3 inflammasome [107,111,112,113]. Pro-IL1 β is then further cleaved by caspase-1 [113], which is activated by assembled inflammasome complexes. Treatment using an unspecific inhibitor (carbenoxolone) in THP-1 monocyte cell line identified that the pannexin-1/endogenous ATP/P2X7 axis is sufficient to induce synthesis and secretion of IL1 β in response to TLR2 stimulation [22,114]. Conversely, TLR4-dependent IL1 β release from THP-1 monocytes is not solely dependent on pannexin-1 [22].

Sepsis is a systemic inflammatory reaction syndrome caused by infection and is associated with organ damage remote from primary infection site [9]. In particular, the liver is likely to be damaged in the case of high grade sepsis [115]. Opened pannexin-1 channels release ATP in the extracellular space, increasing damage and lethality during sepsis. In this context, ATP released from macrophages during peritonitis-induced sepsis has deleterious pro-inflammatory consequences ending with liver damage, when not degraded by CD39 ectonucleotidases [116]. In addition, ATP released via pannexin-1 channels from macrophages has been shown to mediate pyroptosis in a P2X7-dependent manner [31]. Pyroptosis is a pro-inflammatory form of programmed cell death associated with endotoxic shock [117]. In the study from Yang et al., the authors show that caspase-11 activation by intra- but not extracellular LPS leads to the assembly of the non-canonical inflammasome, which cleaves and truncates C-terminal tails and therefore activates pannexin-1 proteins [31]. 

#### 4.8.3. Dendritic Cells

ATP is also released from dendritic cells (DCs) via pannexin-1 channels [23]. This process corresponds to an autocrine amplification loop in DCs: ATP present at the injury site interacts with P2X7 receptors on DCs, leading to activation of pannexin-1 channels and mediating further ATP release. The whole process aims to accelerate the speed of migration to the injury site and the reorganization of the cytoskeleton of DCs.

#### 4.8.4. Pannexin-Mediated Intravascular Crosstalk

Pannexin-1 channels have been shown to modulate the function of platelets and endothelial cells during inflammation. In particular, it has been shown that platelet activation, e.g., via shear stress, is mediated by ATP released through pannexin-1 channels [36]. In another study, collagen-induced platelet aggregation was reduced upon pannexin-1 channels blockade using different pharmacological strategies (probenecid, mefloquine and a specific peptide targeting pannexin-1) as well as genetically modified Panx1^−/−^ platelets in vitro [32]. Subsequent activation of P2X1 via pannexin-1 leads then to increased platelet aggregation [32,36]. Vascular endothelium critically modulates the migration of leucocytes to the site of injury. It has recently been shown that TNF α released upon inflammation induces pannexin-1 opening and ATP release from vascular endothelial cells [29]. Apart from TNF α, thrombin was shown to induce pannexin-1 mediated ATP release from endothelial cells in a PAR-1-dependent manner [97]. Extracellular ATP further induces the activation of vascular endothelial cells in a P2Y receptor-dependent manner leading to increased vascular cell adhesion molecule-1 (VCAM-1) expression [118,119]. This step is critical for adhesion and diapedesis of leucocytes, and indeed, purinergic signaling was shown to be critical for macrophage chemotaxis in this context [120]. 

## 5. Conclusions

Purinergic signaling is now accepted as one of the key players during inflammation. We review here the mechanisms mediating ATP release into the extracellular space from inflammatory cells. These mechanisms are critical in various processes, including the recruitment of inflammatory cells to the damaged or infected sites, fine-tuning of cell activation and immune responses or the establishment of adverse effects during chronic inflammation such as scarring and pain. To specifically modulate ATP release mechanisms would represent a novel therapeutic option for many diseases where specific treatment strategies are still lacking.

## Figures and Tables

**Table 1:** Key Publications Describing ATP Release Mechanisms during Inflammation.

Selected Reading				Type	Cells	Mechanisms
First Author	Last Author	Journal	Year			
Kato, Y.	Miyaji, T.	PNAS	2017	Exocytosis	Neurons, microglia, immune cells	Reduction of neuropathic and inflammatory pain by clodronate (inhibitor of exocytosis) in mice [21]
Parzych, K.	Paul-Clark, M.J.	FASEB	2017	Pannexin-1	THP-1 cells	IL1 β secretion from monocytes upon TLR2 stimulation is dependent on pannexin-1/ATP/P2X7 axis [22]
Saez, P.J.	Saez, J.C.	SCI SIGNAL	2017	Pannexin-1	Dendritic cells	Dendritic cells release ATP via PANX1 hemichannels in response to ATP-dependent P2X7 activation. Released ATP amplifies DCs activation in autocrine manner [23]
Wang, X.	Sun, B.	PNAS	2017	Connexin 43	dHL-60	Neutrophils release ATP via CX43 in response to LPS stimulation. MLCK is activated and phosphorylates MLC, leading to chemotaxis stoppage [24]
Zhang, C.	Du, B.	J IMMUNOL	2017	Exocytosis + pannexin-1	RAW 264.7 cells/293 T cells	ATP is released by virus infected macrophages and protects cells-limiting virus replication-via P2X7 and increased IFNgamma production [25]
Brown, I.A.	Gulbransen, B.D.	CELL MOL GASTROENTEROL HEPATOL	2016	Connexin 43	Enteric glia	Upon oxidative stress, enteric glia release ATP via CX43. This mechanism is potentiated by NO. ATP further activates P2X7 leading to neuron death [26]
Qin, J.	Du, B.	J IMMUNOL	2016	Connexin 43	RAW 264.7	TLRs induce increased CX43 expression in macrophages and UDP release. UDP interacts with P2Y6 receptor and induces MCP-1 release [27]
Lim To, W.K.	Marshall, J.M.	PLACENTA	2015	Exocytosis	Endothelial cells (HUVEC)	Hypoxia induces ATP release which leads to vasodilation via an increased synthesis of PGs and NO [28]
Lohman, A.W.	Isakson, B.E.	NAT COMMUN	2015	Pannexin-1	Endothelial cells (HUVEC)	TNF α released upon inflammation induces ATP release from vascular endothelial cells via PANX1 [29]
Chen, Y.	Junger, W.G.	SHOCK	2015	Pannexin-1	PMNs	Hypertonic saline reduces PMNs overactivation by inducing ATP release via PANX1 channels. ATP is degraded to adenosine that interacts with A2a receptors on PMNs [30]
Yang, D.	Núñez, G.	IMMUNITY	2015	Pannexin-1	BMMφ	Intracellular LPS activated caspase-11 cleaves PANX1 which releases ATP. ATP further activates P2X7 receptors ending with pyroptosis [31]
Molica, F.	Kwak, B.R.	J THROMB HAEMOST	2015	Pannexin-1	Platelets	Collagen induces ATP release from blood platelets and leads to platelet aggregation [32]
Calder, B.W.	Yost, M.J.	TISSUE ENG	2015	Connexin 43	HMVEC	CX43 mediated ATP release in HMVEC was decreased upon treatment with a CX43 mimetic peptide (JM2) and FFAs [33]
Csóka, B.	Haskó, G.	FASEB	2015	Connexin 43	-	ATP is released during sepsis and CX43 blocking leads to increased inflammatory cytokines and bacterial load [34]
Ren, H.	Qian, M.	INFECT IMMUN	2014	Exocytosis	Macrophages	ATP is released from macrophages through TLR activation upon stimulation with LPS and Pam3CSK4 [35]
Taylor, K.A.	Mahaut-Smith, M.P.	J THROMB HAEMOST	2014	Pannexin-1	Platelets	Arterial shear rates induce ATP release via PANX1 in vitro, which ATP interacts with P2X1 and leads to platelet aggregation [36]
Imura, Y.	Koizumi, S.	GLIA	2013	Exocytosis	Microglia	Stimulation with ionomycin or LPS induces release of ATP from microglia by increasing VNUT-dependent exocytotic mechanisms [19]
Sakaki, H.	Kojima, S.	PLOS ONE	2013	Exocytosis	THP-1 monocytes	LPS induced ATP release leads to autocrine P2Y11 activation, M1 polarization and cytokines secretion [37]

## Abbreviations

ATP	Adenosine 5′-triphosphate
BMMф	Bone marrow macrophages
cGMP-PKG	Cyclic guanosine monophosphate-protein kinase G
DCs	Dendritic cells
fMLP	*N*-formyl Met-Leu-Phe
LPS	Lipopolysaccharides
MCP-1	Macrophages chemoattractant protein 1
Nlrp3	NOD-like receptor family, pyrin domain containing 3
NEM	*N*-ethylmaleimide
NFκB	Nuclear factor κ B
PAR-1	protease activated receptor 1
PMф	Peritoneal macrophages
PMN	Polymorphonuclear neutrophil
SNARE	Soluble *N*-Ethylmaleimide-sensitive factor attachment protein receptor
TLR	Toll-like receptor
UTP	Uridine 5′-triphosphate
VCAM-1	Vascular cell adhesion molecule 1
VNUT	Vesicular nucleotide transporter
